# Semantic analysis and the evolution towards participative branding: Do locals communicate the same destination brand values as DMOs?

**DOI:** 10.1371/journal.pone.0206572

**Published:** 2018-11-07

**Authors:** Mohammed Jabreel, Assumpció Huertas, Antonio Moreno

**Affiliations:** 1 TAKA (Intelligent Technologies for Advanced Knowledge Acquisition) research group. Departament d’Enginyeria Informàtica i Matemàtiques, Universitat Rovira i Virgili, Tarragona, Spain; 2 Departament d’Estudis de Comunicació, Universitat Rovira i Virgili, Tarragona, Spain; 3 Computer Science Department, Hodeidah University, Hodeidah, Yemen; The University of Queensland, AUSTRALIA

## Abstract

Participative branding is a process by which DMOs, locals and visitors contribute, through their activity in social media, to the definition of the emotional values associated to a destination. From the communicative point of view it is particularly important that DMOs and local citizens work together in the transmission of a set of coherent values that compose a personalised identity. This paper presents a novel methodology, based on semantic similarity measures, that permits to make an automated analysis of the emotional values transmitted by official tourist offices and by local citizens through social media. A study of 54,000 tweets from 9 main European tourist destinations highlights the lack of a strategy towards the communication of a distinctive brand and a strong gap between the official view transmitted by the DMOs of the destinations and the one communicated by their residents.

## Introduction

The concept of *destination brand*, that represents a unique combination of destination characteristics and added values, both functional and non-functional, which have taken on a relevant meaning [1] and try to convey a favorable image [2], has been heavily studied in tourism and marketing. The research works that have been conducted on this subject have followed two main directions. On the one hand there is *emotional branding*, which highlights the importance of brand emotional values in the communication of tourist destinations [3–7]. On the other hand, a more recent trend is the study of *interactive and participatory branding* [8–11].

*Emotional branding* is based on the idea that many tourist destinations have similar attractions, so at the end it is the emotional aspect of the brand, its personality or identity, that distinguishes it [2, 4, 12–16]. The great contribution of destination brands is the creation of emotional ties with both locals and visitors, which are generated through the relationships they establish with them [17, 18]. These relationships and emotional ties created by destination brands have a key influence in the tourists’ decisions about the places to visit [1]. Thus, *Destination Management Organisations* (DMOs), which are organisations that promote places in order to increase their number of visitors and develop their marketing and services, are posed a double challenge: to communicate the emotional values that make up the identity of the destination and their brand to distinguish themselves from the rest, and to establish good emotional relationships with the tourists in order to create a good image and appeal.

The technological evolution and the emergence of social media have enabled the appearance of *User Generated Content* (UGC), which has provoked an evolution towards *participatory branding* [19]. The communication of a brand, traditionally seen as a unidirectional process, is now understood as a process of interactive dialogue between local and external actors [20, 21]. In particular, their participation in the creation of destination brands is crucial [11, 19, 22, 23]. Thus, DMO managers must create the appropriate interaction channels and encourage their participation in the branding process, especially that of internal or local users [9, 19, 24].

The impact of social media in the communication of destination brands has been enormous. When a tourist visits an unknown place, the word-of-mouth of other users with no vested interest in the destination generates substantial credibility and reduces the risks in the decision making process [7]. Several studies have confirmed that the opinions and experiences shared by users through social media are perceived as being more reliable than the institutional information provided by DMOs of destinations [25–28].

On the basis of these studies it can be said that users, and especially the local ones, play a key role in the branding of destinations. Locals are directly affected by brand decisions and the touristic strategies of the region. Hence, they ought to participate in the branding process. In addition, they are important ambassadors and communicators of this brand and, therefore, their involvement in the entire branding process is crucial [24, 29]. So, the participation of locals is necessary to create sustainable, consistent, non-artificial and successful brand images [10, 29, 30]. However, in professional practice, locals do not always participate in the creation of the brand [9]. In fact, only a few brand creation processes involve them [2, 31].

Given this scenario, the main aim of this paper is to present a methodology that permits to analyse in an unsupervised, automatic and efficient way the brand communicated by a given destination and to compare it with the brand transmitted by its locals.

The analysis of the communication of emotional values through social media presents two main challenges. On the one hand they are intangible and abstract, so their analysis is complex and the researcher may introduce a subjective bias even if specific analysis templates are used to measure them. On the other hand, the amount of messages and comments about a given destination may be huge, making a manual analysis unfeasible. To overcome these limitations this paper introduces a new automated semantic analysis methodology that enables the efficient and objective analysis of a large number of tweets.

Thus, the contributions of this article are twofold. Firstly, it provides a new semantic analysis methodology that allows analysing the communication of brand emotional values posted by DMOs and by their locals, through the analysis of adjectives used in tweets posted by the official destinations and by their residents. Secondly, the proposed methodology permits to find out if the DMOs and locals communicate a coherent brand of the destinations, which will allow us to know if DMOs involve locals in the branding process and if a coordinated participatory branding strategy exists. We understand DMOs and locals have different interests and images of destinations and they will not talk about the same issues in their social media, but it is important that all of them communicate a coherent or, at least, a non-contradictory image of the place. The research is needed by DMOs because would help them to know which image of the destination is communicated by tweets of locals in comparison with theirs.

The rest of the paper is structured as follows. The next section comments the state of the art on participative branding and on the semantic analysis of social media content. After that, the emotional values model is presented. Section 4 describes the methodology used to retrieve, process and semantically analyse a set of tweets. Then, the results of an analysis of 54,000 tweets from 9 major European destinations are presented and discussed. The paper closes with some conclusions and lines of future work.

## 1 Literature review

This section is divided in two parts. The first one discusses two main research trends on collaborative destination branding and their impact on the brand management activities of DMOs. The second one introduces the main lines of work in semantic analysis of social media content.

### 1.1 Participative branding, co-creation of the brand and implications for tourist destinations

Social media have transformed not only the communication of destination brands, but also their definition, turning it into a process involving multiple *stakeholders* (all the publics that are impacted by and also impact a destination, like organisations, politicians, companies, locals, tourists, mass media, etc.) [19]. In fact, several authors claim that one may speak of the existence of a new conceptualization of destination branding, which requires the co-creation of destination brands by all these actors [9, 17, 23, 32, 33]. Two types of branding strategies, focused on shared participation and on co-creation, are commented in the following subsections.

#### 1.1.1 Participative branding studies

At the present moment of technological transformations and social changes, academic studies are evolving towards relational, interactive and participatory branding in the fields of marketing [21, 34–36], public relations [20, 37, 38] or tourism [8–11, 17, 19, 39, 40].

These studies request the participation of locals in the whole process of regional brand creation. They argue that only if relations are struck up with locals and they are involved with the brand of the destination will they accept it and will a sense of identity and of belonging to the destination and to the brand be aroused in them [9].

Participation by locals involves the expression of different points of view on a region, often with conflicting interests; thus, it requires dialogue, disagreement, debate and also negotiation, that is, the establishment of interactions and relationships [10]. The creation of the brand, therefore, must be a democratic process involving the locals and taking their interests into account [3, 41, 42]. Only with the involvement of locals, residents and organizations, who are the ones who really know the region, can a consistent and successful destination brand be defined [43]. Such a commonly agreed-upon brand generates a sense of place among the locals, facilitates their acceptance, and transmits authenticity to tourist.

Despite the wealth of academic publications on the importance of participatory destination branding, in their professional activities DMOs are still assimilating the changes in communication and they are gradually adapting to new technologies and to the idea of participatory branding. Tourist destinations still communicate their brands through slogans and advertising campaigns and they hardly involve their locals in the branding process [2, 19, 31]. Thus, DMOs need to adapt quickly to this new reality, to rethink the role that locals should take in the whole process of destination branding and to create the channels that allow their participation.

#### 1.1.2 Co-creation branding studies

Interestingly, in parallel with these tourism marketing researchers who stress the need of local participation in destination branding, another school of authors from the field of general marketing argues that all the stakeholders are already involved in the creation of brands via their comments in social media [21, 34–36], so their participation in the co-creation of brands is already an inevitable reality. Despite being apparently contrary visions, they are complementary and they move in the same direction. While the latter claim that stakeholders, and locals among them, are already co-creators of the brand via their comments, the former affirm that DMOs should involve locals and make them participate from the beginning of the branding process.

These latter researchers affirm that there has been a great transformation in the field of brand communication. In recent years, many studies have been conducted on communication disciplines concerning brand-consumer relationships [44, 45]. Consumers are currently very active and, furthermore, they are expected to be even more so in the future [46, 47]. They have already begun to participate in the definition of products and their brands, co-creating them on the basis of their comments and experiences. This process leads to a greater implication with the brand, a greater loyalty towards it, a better image and a positive influence on other potential consumers [21].

#### 1.1.3 Implications of these studies for tourist destinations

Thus, a brand is no longer owned by an institution that defines it to convey certain preestablished emotional values in its own interest, but it is created on the basis of all the interactions and relations created in the minds of the stakeholders. Therefore, we may speak of a collective process of brand co-creation [9]. All stakeholders of a brand, from internal/local to external/consumers, are active co-creators of the brand.

This process has also happened in tourism. DMOs have lost the control over the destination brands, which have become the property of a community or a collective. Thus, the meaning of the brand of a destination is constantly being co-created by the participation and comments of the locals, companies, visitors, etc, and, as a result, brands are now more dynamic, authentic and collective [11, 48].

DMOs must bear in mind that the image of a destination in the minds of potential visitors is not only the result of the destination’s communication and advertising campaigns. This situation is due to the emergence of social media. Their capacity to allow users to interact and to create dialogues has transformed branding. Moreover, due to the ease of using social media to share experiences, users have become co-creators of *brands* [34]. Most interestingly, it has been shown that the content generated by users through social media have far greater impact and credibility than those broadcast through traditional channels [49]. Therefore, the co-creation of content by users cannot be ignored by DMOs.

The importance and influence of user-generated content through social media in the creation of destination brands have been previously demonstrated [50, 51]. The images of tourist destinations are created from the interactions and the information of DMOs, locals, other tourists, etc. Social media have become the primary sources of information for potential tourists, and UGC generates information, experiences and emotions that create the image of the brand [52–57].

So, the reality is that whether locals are involved early in the process of brand creation or not, they are always co-creating and communicating the destination brand in any case. Then, it is better to have them involved from the outset. Moreover, DMOs should constantly analyse UGC to know what image of the destination is being communicated by locals, and consequently manage the brand coherently [57].

In the particular case of the work reported in this paper, Twitter was chosen as the social network of study. Many authors have highlighted the increasing importance of social media in tourism information search [50, 58, 59] and, as [60] have shown, Twitter is the fourth main social medium where users search for information, following Tripadvisor, tourism blogs and Facebook. However, the aim of this work is not to analyse how users look for information in the social media when they are organizing a trip, but rather to think about how the comments and experiences that DMOs and locals of destinations share through social media influence in the process of branding co-creation. DMOs should take into account that the image of destinations is not created only by the rational, practical, tangible information that tourists search when they prepare a trip. It is actually created by any content (photographs, videos, emotional comments) made by individual people at any time, based in their own experiences and emotions or those of other users. Moreover, DMOs should also keep in mind that experiences shared by friends and acquaintances are more trustworthy and have more impact in the creation of image destinations than those that are impersonal [61–63], and Facebook and Twitter are currently the most prominent social networks to receive constant information, updates and comments from family and friends. Furthermore, they are also the two social media more commonly used by DMOs to promote destinations. The main advantage of Twitter over Facebook, which led to its final selection, is the sheer amount of messages that are created daily and the capacity to obtain easily messages with given characteristics (e.g. tweets sent from a specific location in a certain language at a particular point of time by users with given characteristics). In any case, the methodology of analysis described in this paper could also be applied to messages distributed through Facebook or any other social network.

### 1.2 Semantic analysis to measure emotional branding

Emotional branding is causing a substantial change in destination management and communication. Destination branding involves the association of distinctive attributes and emotional values to the regions [3, 5, 29]. Therefore, DMOs try to communicate an identity, a personality and a set of values that have an impact on the emotions of the users and generate an attraction for the destination [2]. Destination brands create relationships with stakeholders, whether local or external, which lead to emotional bonds with them [18]. In addition, tourists base their decisions to visit a destination rather on emotional aspects and ties than on rational decisions based only on the main attractions [4].

The images about a place in the minds of potential tourists have a decisive influence on the choice of destination [64, 65]. Many factors influence on the creation of these images, including the expected experiences of the potential tourists, the information received from social media and the behaviour of their locals among others [6, 66, 67]. Therefore, DMOs, with their communicative actions through the various media, seek to transmit an identity and a brand, with the objective of promoting a desired brand image that also is coherent with the vision of the locals [29].

This paper seeks to analyse whether DMOs communicate via their official tweets the same brand and the same emotional values as the locals of destinations. A new methodology of semantic analysis has been designed and developed with this purpose. To date, two types of methodologies have been used to analyse the communication of the emotional content of a destination brand: *qualitative* and *quantitative*.

Qualitative studies are based on methods such as in-depth interviews and focus groups. They provide high quality information, but they are necessarily limited to small samples, and therefore they are not representative of the entire universe. Thus, despite the wealth of information, the results cannot be easily generalized [68]. Many of these studies use qualitative analysis software such as ATLAS.ti or NVIVO. For example, NVIVO was used to analyse how a series of stimuli change the attributes associated with a sample of North American destinations [69], and ATLAS was employed to analyse qualitatively 20 on-line articles about Portugal, in order to ascertain what image tourists have of this destination and how they can help create the destination brand [70]. Another recent example of a qualitative study is the use of semi-structured interviews to determine the use and impact of social media on destination communications [71].

Regarding quantitative studies, they can be mainly classified in two types: *thematic* and *semantic* [72]. Thematic analyses involve the recording of issues, attributes, values or categories by counting the frequency of the words that appear in the text. They permit to know which are the topics or categories that are most relevant in texts, but they are not able to interpret their meaning. Thus, we cannot know if positive or negative things are being said of these items, if there is a touch of irony in the text, what is said precisely about them, etc. Despite these limitations, the measurement of the frequency of words has been heavily used in content analysis. Some recent examples include the analysis of the attributes communicated and associated with the Portugal brand by Russian visitors [13], of the brand attraction factors and emotional values conveyed through social media by DMOs in a sample of Spanish tourist destinations [7] and of the destination brand communication through tourists’ comments in social media [73].

The main contribution of semantic analysis to content analysis is the discovery of the relationships between subjects or categories, creating a more conceptual study rather than a merely syntactic one. Therefore, it can go deeper into the meanings of the words and understand more profoundly what is being said. Just to cite some recent examples, semantic analysis have been employed to assess how the brands of the destinations of Brazil and Mexico are communicated through Facebook [74], to define a “semantic network” to analyse the relations between topics [75], to measure the image of the Basque Country, taking cognitive, affective and conative aspects into account [76] and to study the emotional qualities in tourists’ UGC through the analysis of their perceptions and experiences [77]. Various authors have combined thematic and semantic analysis in their studies [78–80].

This work belongs to this novel line of semantic studies. We have designed and developed a methodology that allows to analyse the communication of something as subjective as brand emotional values through semantic analysis, avoiding the subjectivity and the bias that a manual analysis might involve. Furthermore, it permits to consider a large volume of data, as thousands of tweets can be analysed at a time. This framework of analysis, which is explained in detail in section 4, could certainly be highly useful in the brand management and communication tasks of the DMOs of tourist destinations. Before describing the methodology of analysis, the following section explains which are the emotional values that we want to measure.

## 2 Emotional Model

The importance of the emotional values and the personality of the destinations in their branding has been stressed by many academic studies [1, 81]. Destinations can be described using human personality values [50]. We have applied a very slightly modified version of the well-known *Brand Personality Scale* (BPS) of Aaker to the analysis of destination brands [82]. It has been shown that personality dimensions have a positive impact on the preferences of potential tourists, as a strong and well-defined personality improves the image of the destination and the intentions to visit it [16].

In this work Aaker’s BPS has been used to analyse the emotional values associated to a travel destination. The five main dimensions of analysis of the personality and the emotional values of a destination are *sincerity*, *excitement*, *competence*, *sophistication* and *ruggedness*. Each of them has been divided into a set of categories, which in turn have been refined into several sub-categories, represented by a set of terms. The whole template of analysis is shown on Table 1.

**Table 1:** Emotional Model.

Emotional value	Category	Subcategory
Sincerity	Down-to-earth	Family-oriented
		Down-to-earth
		Sustainable
	Honest	Calm
		Real
		Traditional
		Honest
	Wholesome	Original
		Wholesome
		Quality of Life
	Cheerful	Happiness
		Sentimental
		Friendly
Excitement	Daring	Trendy
		Daring
		Exciting
		Exotic
		Fashionable
	Spirited	Cool
		Spirited
		Dynamic
		Vital
		Fresh
		Young
		Sensorial
	Imaginative	Unique
		Imaginative
		Creative
	Up-to-date	Up-to-date
		Independent
		Contemporary
	Cosmopolitan	Cosmopolitan
		Tolerant
		Hospitable
Competence	Reliable	Reliable
		Hard-working
		Safe
		Rigorous
	Intelligent	Intelligent
		Technical
		Corporate
		Innovative
	Successful	Successful
		Leader
		Ambitious
		Powerful
Sophistication	Luxurious	Glamorous
		Luxurious
	Charming	Seductive
		Smooth
		Romantic
		Magical
Ruggedness	Outdoorsy	Outdoorsy
		Get-away
		Recreational
	Tough	Tough
		Rugged
		Non-Conformist

## 3 Methodology

Twitter and Facebook are the social media more commonly used by tourism destination managers for their promotion. The short updates sent by users in Twitter (*tweets*, limited to a maximum of 140 characters) provide a rich source for finding out the opinions, feelings and emotions of the public. In this work we have focused on the analysis of English tweets sent by official tourist destinations and by their locals. The final aim is to study how the emotional values defined in the previous section are communicated. The steps followed in the methodology of analysis have been the following:

- *Selection of the destinations to be analysed*. In this step we have chosen a set of well-known European destinations, specifying some constraints on the language they use and a minimum quantity of messages transmitted through social media.
- *Retrieval of the set of tweets*. This phase involves the use of a new tool that permits to retrieve sets of tweets that satisfy certain user-given requirements.
- *Pre-processing of the set of tweets*. This step applies some simple treatments on the content of the tweets to make them easier to analyse.
- *Semantic analysis of the content of the tweets*. This step is the core of the methodology. It uses a well-known ontology-based semantic similarity measure to compare the adjectives used in the tweets with the emotional values defined in section 3.
- *Interpretation of the results*. In the final step we can analyse the obtained results, comparing the performance of the DMOs of the different cities and also contrasting the values transmitted by the official DMOs with the ones reflected in the opinions of their locals.

**Fig 1:**
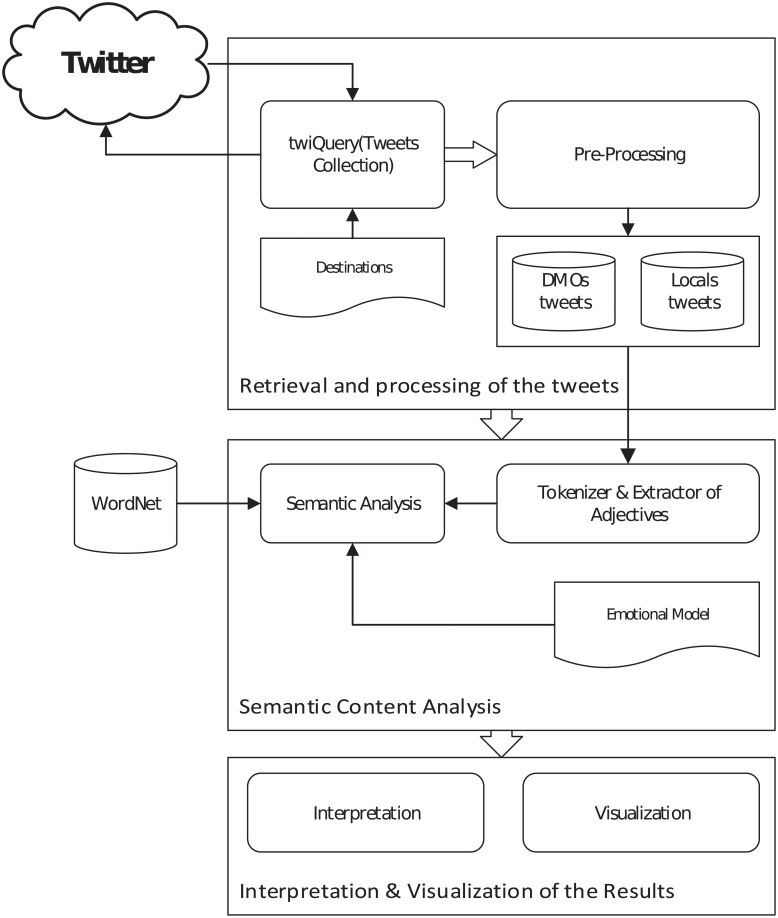
Architecture of the methodological framework of analysis.

Fig 1 shows a graphical depiction of the main steps of this methodology.

In the remainder of this section we describe the technical aspects of the study: how destinations were selected and how their associated tweets were retrieved, pre-processed and semantically analysed.

### 3.1 Destination selection

To select the destinations to be analysed we searched manually for the official Twitter accounts of the top 25 European destinations in 2014, according to Tripadvisor (http://www.tripadvisor.co.uk/TravelersChoice-Destinations-cTop-g4).

In 8 cases (Istanbul, Venice, Florence, Krakow, Urgup, Moscow, Dalyan, and Rimini) we were not able to identify an account. In 5 cases (Rome, Paris, St Petersburg, Madrid and Milan) most of the tweets were written in the main language of the destination and not in English. In some cities (Prague, Lisbon, Zermatt) the number of tweets was very low. It was finally decided to select the nine destinations that had more than 3,000 English tweets, which are shown in Table 2.

**Table 2:** Selection of destinations.

City	Twitter account	Tweets
London	@visitlondon	31,300
Berlin	@berlintourism	5,400
Barcelona	@VisitBCN_EN	5,300
Budapest	@VisitBudapest	3,400
Amsterdam	@Iamsterdam	6,100
Vienna	@ViennaInfoB2B	4,200
Athens	@CityofAthens	4,600
Dublin	@VisitDublin	12,400
Edinburgh	@edinburgh	26,500

### 3.2 Retrieval of the sets of tweets

As we want to compare the communication of the destination brand from the DMO and the local residents’ points of view, in this study we consider two datasets: an *official set of tweets O*, which contains the tweets that have been posted from the official accounts of the selected destinations, and a *local set of tweets L*, which contains tweets posted by the locals of the destinations.

We have developed a novel tool, *twiQuery*, which has been used to collect the tweets to be analysed. twiQuery is a crawler that enables users and developers to make advanced search actions on tweets, such as retrieving sets of tweets posted by a specific user, written in a specific language, sent from a certain area determined by the name of a city or geolocation and a given radius, posted during a specific period of time, etc. The set O is the set of all the tweets written in English and sent by the nine official destination accounts. The set L contains the tweets in English that have been sent within a radius of 15 kms. of the city center of each of the chosen destinations. In both cases the following periods of time were considered:

- High tourism season of 2015: from 15-June-2015 to 01-September-2015.
- High tourism season of 2014: from 15-June-2014 to 01-September-2014.
- Low tourism season of 2014: from 15-October-2014 to 30-December-2014.

In order to make sure that the tweets in the set L have been actually sent by locals, and not by visitors, the system keeps only those tweets that have been sent by users that declare explicitly the destination as their home location in their Twitter profile.

After removing the tweets sent by strangely prolific users (those that have sent over 1,000 tweets in the considered time intervals), we obtained between 11,000 (Budapest) and 370,000 (London) local tweets per each of the nine cities. As we want to compare easily the references to emotional values from locals and from destinations, we finally decided to analyse 3,000 tweets from the official destination account and 3,000 tweets from locals for each of the nine cities (Amsterdam, Athens, Barcelona, Berlin, Budapest, Dublin, Edinburgh, London and Vienna). The tweets were randomly chosen. Table 3 shows the number and percentage of different local users for each destination.

**Table 3:** Unique users in each analysed destination.

Destination	Unique Twitter users	Percentage
London	2555	85%
Berlin	1256	42%
Edinburgh	1419	47%
Dublin	1918	64%
Athens	1012	34%
Vienna	958	32%
Amsterdam	1347	45%
Budapest	781	26%
Barcelona	1333	44%

It has to be noted that, even after filtering the locality of the users, it may not be guaranteed that they are talking about the destination. In order to study this issue, a manual analysis of 400 random tweets sent by local people from London was made. This study showed that 292 tweets (73%) were related to local aspects of the destination (local news, weather, restaurants/food, local transport, tourist attractions and cultural activities, local stores and business, local sports, etc.), 97 tweets (24.25%) were conversational or personal life comments, and only 11 (2.75%) were referring to other locations or global news. In the light of these results, we believe that the set of local tweets is representative enough of the local aspects of the destination (however, in the future work it could be possible to devise more precise ways of filtering the set of local tweets).

### 3.3 Pre-processing of the tweets

It is well-known that the language used in Twitter is very casual and noisy. Tweets contain numerous punctuation errors, spelling mistakes, abbreviations, slang terms, emoticons, etc. The tweets were pre-processed to mitigate some of these effects as follows:

- Tweets may contain URLs, usernames, hashtags and emoticons. All URLs, usernames and strange symbols were removed from the tweets.
- To reduce the dimensionality, all tweets were converted to lowercase and stop words were removed. Table 4 shows the list of stop words.
- Words with repeated letters were automatically corrected using an algorithm devised and implemented by the authors that performs a breadth-first search and analyses all the possible ways of eliminating repeated letters in a string, checking in Wordnet (see http://wordnet.princeton.edu) if they are correct.

**Table 4:** Stop words list.

Stop words list
i, me, my, myself, we, our, ours, ourselves, yo, your, yours, yourself, yourselves, he, him, his, himself, she, her, hers, herself, it, its, itself, they, them, their, theirs, themselves, what, which, who, whom, this, that, these, those, am, is, are, was, were, be, been, being, have, has, had, having, do, does, did, doing, a, an, the, and, but, if, or, because, as, until, while, of, at, by, for, with, about, against, between, into, through, during, before, after, above, below, to, from, up, down, in, out, on, off, over, under, again, further, then, once, here, there, when, where, why, how, all, any, both, each, few, more, most, other, some, such, no, nor, not, only, own, same, so, than, too, very, s, t, can, will, just, don, should, now

### 3.4 Semantic content analysis

In previous studies on content analysis of the communication of destination brands it was pointed out that *nouns* usually provide information on the particular tourist attractions, *verbs* describe actions and types of tourism, and *adjectives* communicate the emotional responses [78, 83]. Thus, as we want to measure the association between tweets and emotional values, we have focused the analysis on the *adjectives* used by destinations and visitors.

The objective of the semantic analysis is to associate the adjectives found in a set of tweets with the categories of emotional values shown in Table 1. We used the Penn Treebank POS-tagger from the NLTK library [84] to retrieve the adjectives and count their frequency of use. A direct syntactic mapping is not possible, as most of the adjectives do not appear directly as categories/subcategories of emotional values. The idea is to use a *semantic* similarity measure [85] between the adjectives and the categories/subcategories. This type of measures requires the use of some kind of external structured knowledge base (in our case, WordNet). Ontology-based semantic similarity measures rely on the topological structure of an ontology to calculate the degree of resemblance between two terms. The length of the path between the terms (considering hyponymy and hypernymy relationships) and their position in the hierarchy of concepts (i.e. their degree of generality) are the basic points taken into account by similarity functions. In this work we have used a well-known similarity measure, defined by Wu and Palmer [86] as follows:

$$Sim_{W\&P}\left(c1,c2\right)=\frac{2*depth(c3)}{length(c1,c3)+length(c2,c3)+2*depth(c3)}$$ (1)

In this expression *c3* is the *Least Common Subsumer* (LCS) of *c1* and *c2* in the reference ontology, *length* is a function that returns the number of hypernym links among two concepts and the *depth* of a concept is the number of hyperlinks that separate it from the root of the ontology. This measure ranges from 1 (for identical or synonym concepts) to 0 (when the LCS of the concepts *c1* and *c2* is the root of the ontology, so they do not have any common ancestor). The main difference of this function with respect to other edge-counting measures is that it takes into account the depth of the compared concepts in the hierarchy (given the same distance, two concepts are more similar if they are more specific). The semantic similarity between two words is calculated as the maximum similarity between the synsets associated to each of the words in WordNet (which represent their different senses). The adjectives with a similarity higher than 0.7 were considered as *emotional adjectives* and counted in the analysis, whereas the rest were dismissed.

The main problem of this approach is that WordNet uses hypernymy relationships between nouns, and we wanted to compare the adjectives found on the tweets with the subcategories associated to the emotional values (which are also mostly adjectives). Thus, before applying the Wu-Palmer semantic similarity measure we had to transform its inputs into nouns. In the case of the subcategories, they were manually translated to the equivalent nouns (e.g. *friendly* and *ambitious* were transformed into *friend* and *ambition*, respectively). Concerning the adjectives appearing in the tweets, they were automatically transformed into nouns using their *derivationally related form* or their *attribute* property in WordNet.

Table 5 shows some tweets posted by local people from London and analysed using our model. The table shows the tweet (with the analysed adjective in bold face), the emotional value that has been considered more similar to the adjective, and the degree of similarity between them. Note that there may be parsing errors (e.g. “*love*” is labelled as an adjective in the second tweet).

**Table 5:** Example of tweets analysed using our model.

Tweet	Emotional Value	Similarity
Amazing **creative** buildings on #EastLondon #lovelondon @ Shoreditch High Street <URL>	Creative	1.0
Summer **love**. @ St James’s Park <URL>	Romantic	0.93
Another **joyful** day in this amazing city!!! #London #londonpride, #LoveWins #Friends #Pride2015… <URL>	Exciting	0.93
**Beautiful** morning for a ride. #mangobikes #cycling #lovetheweekend @ Botley Hill Farmhouse <URL>	Glamorous	0.92

Let us consider a concrete example, using the last tweet of that table. The adjective “beautiful” is transformed automatically into a noun (“beauty”) using the *attribute* property of WordNet. Then, it can be compared with the nouns that represent the different emotional values. Some results (the interested reader may find in http://ws4jdemo.appspot.com/ a detailed explanation of how these results are obtained after analysing the WordNet noun hierarchy) are the following:

- *Sim*_*W*&*P*_(beauty, glamour) = 0.93
- *Sim*_*W*&*P*_(beauty, charm) = 0.87
- *Sim*_*W*&*P*_(beauty, honesty) = 0.66
- *Sim*_*W*&*P*_(beauty, original) = 0.63
- *Sim*_*W*&*P*_(beauty, innovation) = 0.55

Thus, the system considers that the term *beautiful* is highly related to emotional values such as *glamorous* or *charming*, but it is quite unrelated to the values *honest*, *original* and *innovative*.

The accuracy of the automatic semantic procedure that links adjectives to emotional values is hard to measure, since even a manual assessment is highly subjective. The common procedure to evaluate the performance of a semantic similarity measure is to calculate the correlation between its results and a human assessment of the similarity between some pairs of terms belonging to a golden standard. In the case of the Wu-Palmer similarity, Slimani [87] obtained a 74% correlation and Budanitsky and Hirst [88] report 82.9% and 81.9% in two different benchmarks (using the Lin measure, of which Wu-Palmer is a particular case [89]).

## 4 Results and discussions

In Tables 6 and 7, which present the results of the analysis of the communication of the brand emotional values by DMOs and by locals or residents of destinations through their tweets, two figures appear for each emotional value and destination. The first one is the total number of times that an emotional value has been communicated (possibly using a certain number of adjectives), whereas the second figure is the number of different adjectives used to communicate an emotional value in the analysed tweets. For example, the official tourist account of London used 9 adjectives 33 times to convey the *calm* value. The first row shows the accumulated values for each city (e.g. Berlin locals have used 186 emotional adjectives 1086 times in their 3000 tweets). The nine columns in the left part of the figure correspond to the analysis of the tweets sent by the official tourist accounts, whereas the ones on the right side provide the results of the analysis of the tweets sent by local people. The orange cells highlight the positions with higher values. Green cells mark situations in which a city does not communicate a value that is transmitted by most of the other destinations (for example, Dublin’s DMO is the only one that did not communicate the value *wholesome*). Yellow cells indicate a value that is transmitted by a low number of destinations (e.g. the local people from Athens were the only ones that referred to the *cosmopolitan* concept, albeit only once).

**Table 6:** Use of emotional adjectives by DMOs (orange: high values—green: usual emotional value not communicated by the destination—yellow: emotional value communicated only by a few destinations).

Subcategory	Lond	Berl	Dubl	Athe	Edin	Vien	Amst	Buda	Barc
#	1103/261	945/222	1011/245	840/219	1082/266	893/206	943/229	885/199	760/192
Family-oriented			3/1	2/2				1/1	
Down-to-earth	57/25	55/25	60/22	52/20	54/19	44/18	48/25	64/24	43/22
Sustainable	6/6	7/6	4/2	8/7	9/8	3/3	3/3	1/1	3/1
Calm	33/9	30/7	33/7	23/6	37/8	24/9	28/7	18/6	26/9
Real	39/6	31/8	28/8	32/6	31/7	25/5	27/6	32/7	26/6
Traditional	17/10	14/9	18/11	21/10	9/9	15/10	11/7	12/7	9/8
Honest	162/10	141/4	163/9	150/7	184/11	141/7	163/8	145/6	147/6
Original	45/26	43/20	37/21	33/17	37/20	25/11	26/14	24/10	32/11
Wholesome	6/1	6/1		3/1	4/1	2/1	7/1	7/1	6/1
Quality of life									
Hapyness	31/8	28/8	36/7	39/8	48/12	35/8	24/7	57/7	32/6
Sentimental	27/13	21/9	22/14	18/13	19/11	19/12	16/11	24/14	14/11
Friendly	3/3	6/3	11/4	5/4	9/4	1/1	3/2	4/3	12/6
Trendy	2/2	1/1	5/4	4/2	6/4	1/1	2/2	1/1	2/2
Daring	22/7	17/4	17/6	9/7	11/5	14/5	14/5	10/4	12/7
Exciting	6/2	4/2	5/1	7/2	2/2	6/2	4/1	1/1	3/1
Exotic	1/1		2/1			3/2	2/2	6/2	1/1
Fashionable						1/1			
Cool									
Spirited	4/3	1/1	1/1	2/2	3/2		4/2	3/2	6/2
Dynamic	1/1	1/1		1/1	1/1	2/2	2/2	2/2	2/2
Vital		1/1	2/1						
Fresh	156/7	143/5	94/5	109/7	134/6	129/5	158/4	91/8	98/4
Young	12/3	11/2	7/2	6/2	4/1	2/2	5/1	8/2	5/1
Sensorial	20/6	25/7	34/9	16/5	23/12	25/8	25/7	20/7	8/3
Unique	23/4	9/2	12/2	19/3	19/5	9/2	13/4	11/3	15/2
Imaginative					1/1				
Creative	14/6	10/5	11/5	11/6	6/3	10/6	16/6	6/2	3/1
Up-to-date	3/2	2/2	4/1	2/1	4/2	7/2	1/1	2/2	7/2
Independent	2/2	3/2	2/2	1/1	7/2	5/1	1/1	6/2	3/2
Contemporary	57/9	60/9	67/10	40/9	64/8	78/9	45/9	82/7	25/5
Cosmopolitan					1/1	1/1			
Tolerant	2/1		1/1			1/1			2/1
Hospitable	1/1		2/2	1/1		2/1	1/1	3/1	2/1
Reliable	2/1		2/1	1/1	2/1		1/1	2/1	
Hard-working						4/1		1/1	1/1
Safe	57/15	43/11	37/15	22/8	46/15	23/10	31/14	31/12	26/11
Rigorous	1/1	1/1					1/1	1/1	
Intelligent	11/6	9/7	12/4	5/5	16/8	6/4	11/6	1/1	3/2
Technical	2/2	2/2	2/1	1/1	5/3	1/1	1/1	1/1	3/1
Corporate	3/1	3/2	4/3	4/2	1/1	8/3	7/1	3/2	3/2
Innovative	7/3	20/4	6/2	9/2	10/5	8/3	14/3	7/3	4/1
Successful	5/3	2/1	5/2	6/2	8/2	6/2	4/3	3/2	1/1
Leader	66/16	61/9	73/14	54/12	72/16	71/13	65/15	57/11	49/12
Ambitious	4/2	1/1	7/4	4/2	6/3	1/1	2/2	1/1	5/4
Powerful	2/2	16/6	11/3	7/3	12/5	4/3	12/4	7/3	5/2
Glamorous	66/6	41/6	39/6	44/6	44/6	58/5	41/4	42/6	33/4
Luxurious	9/3	5/2	10/3	4/3	14/4	7/2	6/3	3/2	6/3
Charming	1/1		3/2	1/1	4/2	1/1	1/1		1/1
Smooth	22/2	22/3	21/4	12/5	19/5	23/4	24/6	19/3	17/2
Romantic	5/1	3/1	4/1	11/1	3/1	7/1	8/1	6/1	9/1
Magical	12/2	9/2	8/1	5/1	20/2	1/1	6/2	12/1	2/1
Outdoorsy									
Get-away	7/5	7/5	8/5	9/4	10/6	4/4	13/7	5/3	7/6
Recreational	3/2	2/1	4/1	4/1	4/2		4/2	1/1	3/2
Tough	2/1		3/1	1/1	1/1	1/1	2/1	1/1	2/1
Rugged	60/10	22/10	60/9	20/7	52/10	26/8	38/11	36/8	32/9
Non-conformist	4/2	6/4	11/4	2/1	6/3	3/2	2/1	4/1	4/1

**Table 7:** Use of emotional adjectives by locals (orange: high values—green: usual emotional value not communicated by the destination—yellow: emotional value communicated only by a few destinations).

Subcategory	Lond	Berl	Dubl	Athe	Edin	Vien	Amst	Buda	Barc
#	3565/289	1086/186	923/153	1495/197	1140/162	1251/202	1193/192	699/143	1054/171
Family-oriented		1/1						3/1	1/1
Down-to-earth	118/22	53/15	15/8	90/16	66/13	84/19	50/18	41/14	39/15
Sustainable	15/9	12/4	1/1	12/4	2/1	8/1	9/4	7/4	4/2
Calm	127/9	73/4	29/6	52/4	68/5	59/4	67/4	40/2	51/4
Real	28/6	18/6	18/5	27/5	10/4	17/5	17/5	9/4	13/4
Traditional	106/21	35/9	7/3	72/12	21/6	50/11	49/13	36/5	21/10
Honest	399/10	163/5	178/7	328/7	146/5	124/8	135/8	104/6	285/5
Original	114/23	42/15	13/10	50/15	16/9	59/19	32/13	19/14	41/14
Wholesome	4/1	1/1	1/1	2/1	5/2		2/1		
Quality of life									
Hapyness	57/6	11/4	8/6	6/4	40/3	22/4	29/4	16/3	15/3
Sentimental	25/10	10/4	12/5	12/8	10/4	8/5	6/4	8/5	3/3
Friendly	20/7	5/3	4/3	5/2	5/3	7/4	17/5	2/1	8/4
Trendy	23/6	13/3	12/2	31/3		18/2	17/3	55/2	39/2
Daring	40/8	13/7	9/4	18/4	19/6	14/6	22/7	13/5	33/7
Exciting	6/1	5/2	2/2	1/1	5/3		9/2	1/1	2/2
Exotic	5/2	4/2		4/1	1/1	2/2	12/2		2/2
Fashionable									
Cool									
Spirited	6/2	1/1	1/1	4/1	7/3	8/3	4/3		4/3
Dynamic	3/3	9/3		1/1	2/2	20/4	4/1	1/1	23/3
Vital			1/1		8/1				
Fresh	1141/6	208/6	70/7	210/6	131/4	224/6	218/6	123/3	56/5
Young	11/2	6/2		2/1	2/1	15/2	5/1	2/1	4/1
Sensorial	38/11	11/4	66/10	52/5	20/6	15/6	14/8	9/4	7/5
Unique	124/5	25/4	14/3	33/4	41/4	42/5	53/3	20/4	36/5
Imaginative						1/1			
Creative	29/7	35/6	10/5	21/7	3/3	20/7	13/2	4/3	4/4
Up-to-date	30/2	7/2	3/1	27/2	1/1	50/2	11/2	12/2	20/2
Independent	12/3	2/2	7/2	5/1	1/1	1/1	1/1		2/2
Contemporary	333/9	63/9	42/5	32/10	71/9	60/7	43/7	31/6	45/5
Cosmopolitan				1/1					
Tolerant			1/1	1/1				1/1	
Hospitable	1/1	1/1		5/2			2/1	1/1	
Reliable							1/1		
Hard-working	1/1		1/1		1/1				
Safe	63/14	21/9	39/8	56/11	24/10	51/12	40/11	20/8	37/9
Rigorous		1/1							3/1
Intelligent	23/7	22/8	8/5	23/5	7/4	11/3	30/6	3/3	16/3
Technical	9/1								1/1
Corporate	1/1	1/1	1/1	1/1	2/2		4/1	1/1	
Innovative	28/3	7/1	22/2	10/3	14/3	10/3	13/4	6/2	5/2
Successful	3/2	5/2		5/2	11/2	7/3	8/3	7/2	1/1
Leader	125/20	34/9	37/6	67/21	34/10	82/12	64/11	35/16	76/11
Ambitious	4/3	2/1	1/1		1/1	2/2	1/1		5/4
Powerful	17/4	12/4	10/3	14/4	8/3	7/2	9/4	1/1	3/1
Glamorous	76/6	49/2	105/5	89/2	111/6	58/4	93/4	17/4	33/4
Luxurious	7/2	6/3	2/2	1/1	7/2	7/3	2/1	1/1	5/2
Charming					2/1	4/1	1/1	1/1	1/1
Smooth	17/4	9/3	18/2	28/3	8/2	12/5	6/2	11/2	46/3
Romantic	3/1	2/1	5/1	8/1	3/1		2/1		2/1
Magical	105/2	9/1	69/2	14/1	108/2	13/1	9/2	1/1	4/1
Outdoorsy									
Get-away	64/11	8/4	9/4	15/4	22/5	10/6	11/4	5/3	19/3
Recreational	5/3		3/1	1/1		1/1		2/1	10/2
Tough	1/1	1/1		1/1					
Rugged	192/7	69/9	65/6	58/7	76/7	46/8	56/6	30/4	27/7
Non-conformist	6/4	1/1	4/4			2/2	2/1		2/1

The figures on the right of each cell show that, in general, DMOs use a greater number of different emotional adjectives in their tweets than the locals. This greater number of adjectives could be intentional on the part of the DMOs, because they could be trying to use more adjectives to communicate a value. If we look at the exceptions for particular emotional values, it can be seen that locals use a (very slightly) higher number of adjectives than DMOs just for the values *daring*, *spirited*, *dynamic* and *creative*. If we look at the exceptions for destinations, only the locals of London use a higher number of emotional adjectives than the destination. Therefore, there are very few exceptions indeed.

The leftmost figure in each cell is a more appropriate datum for the evaluation of the communication of the emotional values, because it shows the total number of times that a value is actually communicated. For example, a value can be communicated only with three different adjectives, but they can be used many times. If we look at these figures, with the exception of Dublin and Budapest, locals communicate more emotional values than DMOs. This poses two realities.

First, it may lead one to think that most DMOs of tourist destinations do not actually have an established brand communication strategy. These results coincide with others from previous studies [37, 90, 91] that have analysed various social media and various samples of destinations. All their results show the inexistence of brand communication strategies in the management of social media.

Secondly, if we focus only on the adjectives used in the tweets, these results show that locals or residents are better communicators of emotional values than DMOs. This fact would also coincide with the studies of participatory branding that claim that all relevant stakeholders (including residents) are important brand co-creators [21, 34–36]. Therefore, one of the contributions of this study, which was quite unexpected, is to demonstrate quantitatively a reality that was known, but that was difficult to measure or had not been measured in such a clear and quantifiable way.

### 4.1 Analysis per emotional value

In this subsection we first analyse the number of adjectives used to communicate each emotional value (the right numbers on the cells of Tables 6 and 7). After that, we focus on the total number of emotional values that have been transmitted or communicated by the adjectives employed by DMOs or locals in their tweets (the values on the left on the same table).

Firstly, the numbers in Tables 6 and 7 show that the values communicated by a larger number of different adjectives are virtually the same in the tweets of destinations and in those of locals. Those that use a higher number of adjectives to be communicated are *down-to-earth*, *original*, *secure/safe* and *leader*. These values are indeed interesting both to destinations and locals alike, especially with regard to security/safety, leadership or originality. In theory, all destinations would like to be original and secure/safe. On the other hand, there are emotional values that are hardly communicated by any adjective (or not communicated at all) in the tweets of destinations and locals. It is surprising that values such as *quality of life*, *fashionable*, *cool* or *cosmopolitan* are not transmitted by any adjective, since they are values with which many destinations would probably like to be associated. This fact will have to be analysed in further detail, since it might be due to a poor choice of the nouns that represent these values in WordNet.

Secondly, if we look now at the values most communicated by tourist destinations and by locals, independently of the number of adjectives used, analysing the left figure of each cell, *honest* and *fresh* stand out, and they are far more communicated by locals than by DMOs. The locals of London, for example, communicate the value *fresh* 1141 times in their 3000 tweets when the destination only communicates it 156 times. This means that the locals of London truly have an image of the city to which they associate this value and the DMO should take this fact into account in its brand communication strategy.

Other values that are also quite communicated are *down-to-earth*, *calm*, *original*, *contemporary*, *secure/safe*, *leader*, *glamorous* and *rugged*. The majority of them are more communicated by locals than by tourist destinations, although there are some exceptions. The value *traditional*, for example, which is communicated by all destinations with low scores, shows very high scores in the tweets of the locals of London, Athens or Vienna. Thus, the locals of these three destinations consider that their cities are traditional while their DMOs hardly communicate this fact at all. The *glamorous* emotional value is also communicated with medium frequency by all DMOs of the analysed destinations; however, the residents of Edinburgh, Dublin and Amsterdam associate this value with their cities with frequencies that triple those of the destinations. Just to mention another example, the residents of London communicate the *contemporary* value six times more frequently than the destination itself. In these cases, in which there is a distinguishing feature attributed by locals to some tourist destinations, these emotional values acquire a distinctive role of authenticity, because the locals know the identities of the destinations better than anyone. Therefore, the DMOs should be highly receptive to these values and take them into account in the management of the identity and the brand of their destination. Although locals and DMOs can have different images of a destination, it is important to guarantee a certain level of coherence between them, which will help to build a strong brand image and a successful positioning.

The opposite situation appears in the value *happiness*, that is communicated with average scores by all of the DMOs whereas it is hardly communicated by the locals of Athens and Dublin. In the case of Athens, the severe economic crisis the country has suffered during the last few years added to the current immigration problems may have been the reasons why the locals of Athens do not associate happiness with this city, because the score is really very low compared to those of the locals of the rest of the analysed cities.

In some cases it may be seen that a destination communicates an emotional value much more frequently than the locals (e.g. the *contemporary* value in Budapest). This effect probably occurs when a DMO aims to promote an image that is desired but does not correspond fully to reality. Instead, locals offer a far more real image of the city.

### 4.2 Analysis per destination

If we now analyse the number of adjectives that communicate emotional values in each destination, it may be noted that the DMOs that use the highest number of adjectives are Edinburgh, London and Dublin (These are the English-speaking destinations in the dataset. The effect of the native tongue of the destination should probably be studied more carefully). Conversely, the locals who use most adjectives are those of London and Vienna. However, as said before, these figures only show a communicative intention, but they do not reflect all the communication of emotional values.

So, now we focus on the first figure of each cell, which shows the total number of times an emotional value has been communicated. In this case, the DMOs that communicate more emotional values with their tweets are again London, Edinburgh and Dublin, in this order, and the locals that communicate more values are those of London, Athens and Vienna. The locals of London triple the values communicated by the London DMO and those of Athens double the values of the destination. Thus, it seems that the DMOs of these three destinations (London, Edinburgh and Dublin) have a communication strategy through Twitter. Now we will analyse which are the values they communicate, whether they are differential values, and if they are shared by their locals.

London communicates the values *honest* and *fresh* with high frequencies, and *down-to-earth*, *original*, *contemporary*, *leader* and *rugged* with medium frequencies. All these emotional values are the most communicated by almost all major tourist destinations. Therefore, the existence of a differentiated brand communication strategy that aims to separate the destination from the rest and identify it with original values is not apparent. However, the locals do communicate emotional values with very different frequencies from those of the DMO and other destinations. For example, the locals of London associate the destination with the value *rugged* with a frequency that triples the one of the rest of cities and that of the DMO. This does seem to be a characteristic value of the destination according to its locals. The same thing happens with the values *leader* and *original*.

The Edinburgh DMO, for example, also communicates the same generic emotional values than the rest of DMOs. Therefore, it does not show either a strategic and differentiated brand communication strategy. However, the tweets of locals do communicate differentiating values. For example, *calm* is communicated with average frequency, but locals double this frequency. They also double the frequencies of the values *glamorous* and *unique/diverse*. Thus, they communicate that their city is quiet, unique, diverse and glamorous. On the other hand, the DMO communicates the values *secure/safe* and *leader* with medium and high scores and the locals show frequencies much lower than those of the DMOs. Thus, the locals of Edinburgh do not communicate that their city is safe or a leader. The DMO should take all of this into account and analyse whether it communicates the reality of the territory, try to solve the existing problems, reposition the identity and the brand of the destination and then communicate these values powerfully.

Something very similar is seen in the communication of emotional values by the city of Dublin. The values communicated by the DMO are those shared by most destinations. However, the different values communicated by its locals distinguish the territory. The DMO communicates with medium-high frequencies the values *down-to-earth* and *contemporary* but its locals communicate these values with low frequencies. In contrast, the DMO communicates *sensorial* and *glamorous* with medium-low frequencies whereas locals communicate these values with high frequencies. Thus, the locals of Dublin communicate differential and unique brand values while the DMO merely communicates undistinguishing values.

**Fig 2:**
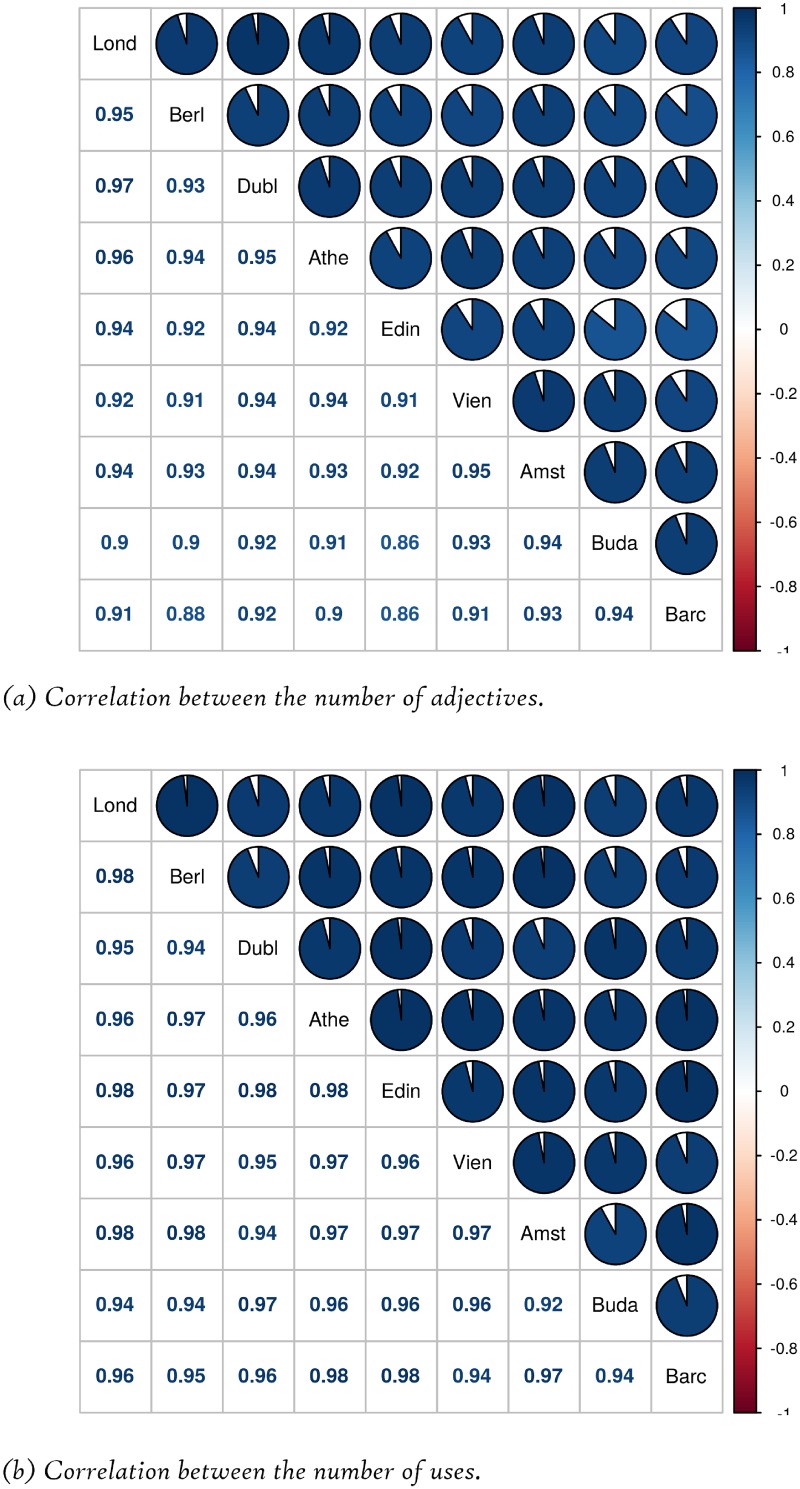
Correlation between the data of each DMO.

We have also measured the Pearson correlation between the data of the DMOs for each emotional value, studying in a separate way the number of adjectives and the total number of uses of the adjectives. The results are shown in the symmetric matrices depicted in Fig 2 in a graphical and a numerical form.

It may be seen that the correlation between the number of adjectives used for each emotional value is quite high (between 0.86 and 0.97), whereas the correlation between the total number of uses of the adjectives is even higher (between 0.92 and 0.98). Thus, it may be concluded that there are not very significant differences between DMOs in this respect.

### 4.3 Comparison between DMOs and locals

**Fig 3:**
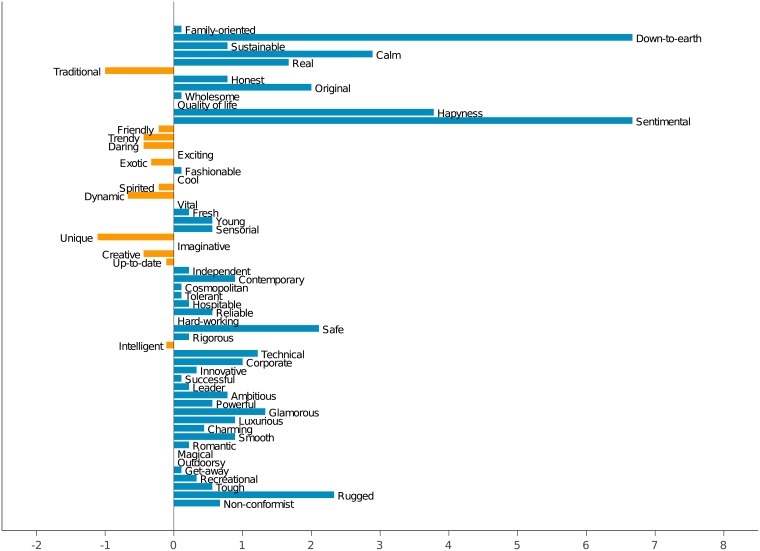
Difference on the number of emotional adjectives between DMOs and local citizens.

**Fig 4:**
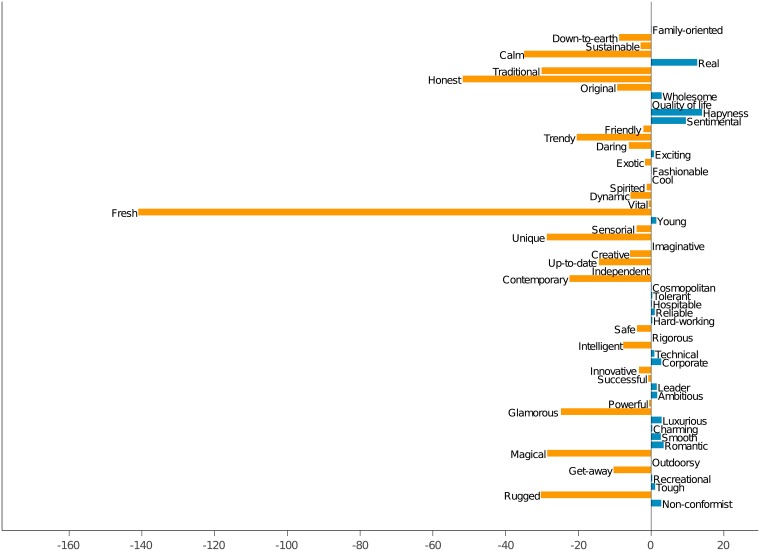
Difference on the uses of emotional adjectives between DMOs and local citizens.

Figs 3 and 4 provide a graphical representation of the difference in the communication of emotional values among DMOs and local people. The first one shows, for each emotional value, the difference between the average numbers of adjectives used by DMOs and by locals. It is easily seen that, in the majority of cases, DMOs use more adjectives, being *down-to-earth*, *sentimental*, *happiness* and *calm* the most prominent cases. There are only a few values for which DMOs have a slightly higher number of emotional adjectives, being *traditional* and *unique* the top ones.

In turn, Fig 4 makes the same analysis but with the difference on the average number of total uses of adjectives referring to each one of the emotional values. As commented before, in this case the superiority is on the DMO’s side, very specially in the *fresh* concept. Other values heavily mentioned by DMOs include *honest*, *calm*, *traditional*, *unique*, *rugged* and *magical*. On the other hand, there are a few values more mentioned by locals, in special *happiness*, *real* and *sentimental*.

**Fig 5:**
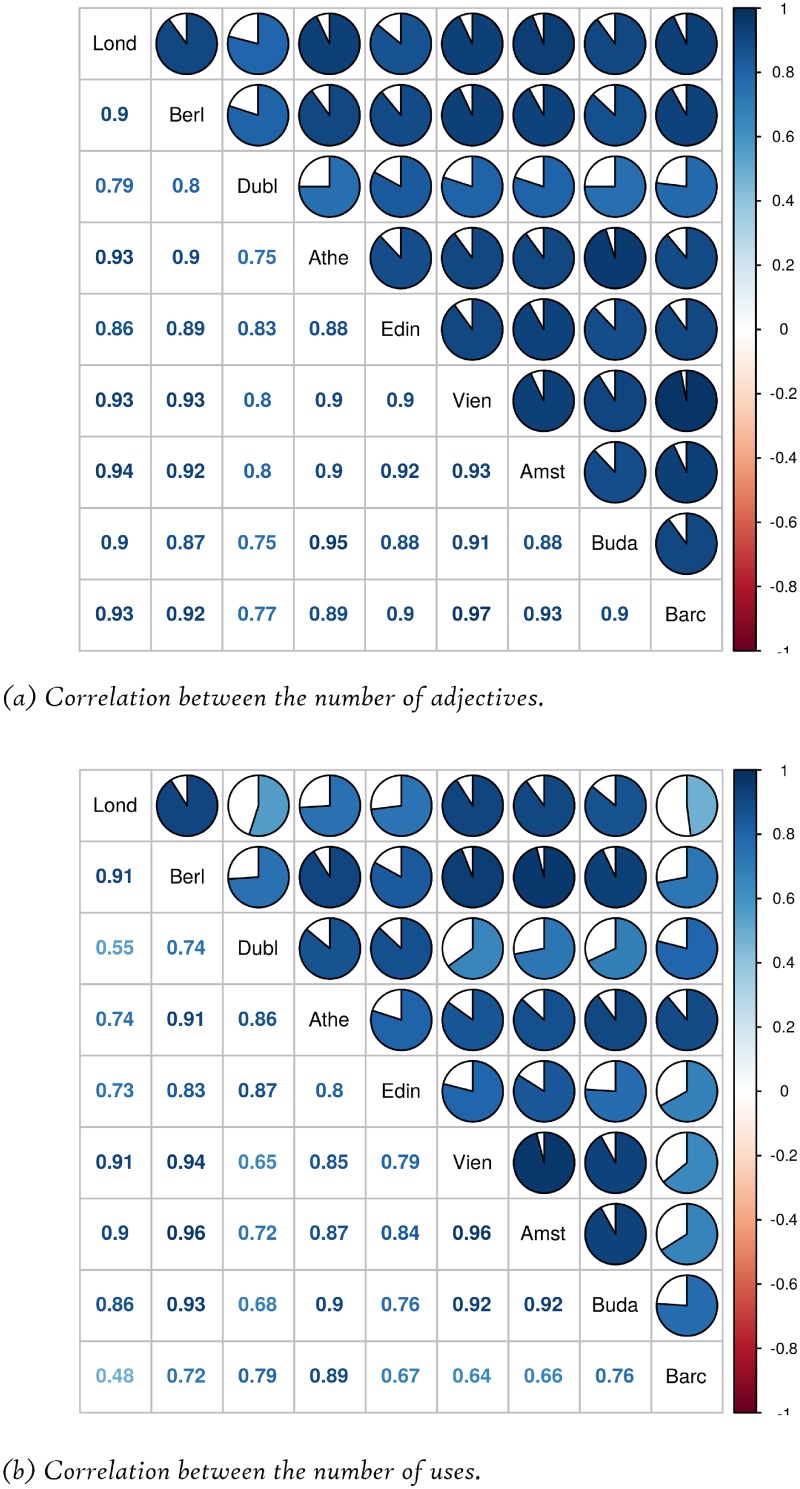
Correlation between the data of local residents.

As in the case of DMOs, we also measured the Pearson correlation between the different cities, taking into account the number of adjectives and the uses of those adjectives for each emotional value. The results are shown in Fig 5. The differences among cities are much more significant than in the case of DMOs commented in the previous section. Concretely, the correlation between the number of adjectives used for each emotional value ranges from 0.75 (between Dublin and Athena/Budapest) to 0.97 (between Barcelona and Vienna). The differences are even higher when the total number of uses of the adjectives is considered. In this case, the lowest correlation is 0.48 (between London and Barcelona) and the highest one is 0.95 (between Amsterdam and Berlin/Vienna).

## Conclusions

The results of the study show that DMOs, despite using a greater variety of adjectives, generally communicate less emotional values than locals. In addition, no great differences are observed in the use of these values. That is, the values communicated by all DMOs have a high coincidence. Thus, few distinctive features are observed. As a result, the first conclusion of the study seems to be that DMOs do not have a clearly established brand communication and differentiation strategy. The reasons may be that they prefer to communicate activities and agenda than the brand in their online communication, that they do not focus on emotional values in brand communication, or that they do not establish guidelines for content communication specifying the terms to be used. Other studies have also shown that many DMOs prefer to communicate the tourist attractions or the city agenda than the emotional values and, therefore, values end up being less communicated [7, 90].

So, the first recommendation for DMOs is to take great care of the communication of the destination brand. The brand and the emotional values it associates to the regions are critical in distinguishing the destination from others [2, 4, 12, 14–16] and in creating partnerships and relationships with the locals [17, 18]. There is a need to take care of the communication of emotional values through all communication channels and especially through social media. As mentioned above, these new communication channels have great potential for emotional communication and they should not be underused [50, 92]. Moreover, for the excellent communication of certain emotional values there must be a strategy to communicate specific content, which sets the terms to use in communications through social media. Given that Twitter only allows sharing 140 characters, the communication through this microblog must take extreme care of each of the employed words.

Furthermore, the results show that locals, contrary to what we expected to find, communicate more emotional values than DMOs. In addition, they do so using fewer adjectives, and they sometimes show special differential emotional values that set their destination apart from the rest and are not shared by all destinations. These results, as mentioned above, confirm the results of studies on participatory branding that assert that users are important brand co-creators [21, 34–36]. In fact, another contribution of this study has been to demonstrate that this participation in the on-line creation of a distinctive brand is indeed real and, moreover, quantifiable and measurable. It should also be borne in mind that the comments and the image that locals have of their town/city provide a twofold additional credibility. First, because they know the region better than anyone, and second, because, in principle, they have no vested economic or commercial interest in the association of emotional values to the territory.

All this leads to a second implication of the study for DMOs of tourist destinations. Since locals co-create brands through their comments and experiences in social media, and because their knowledge of the area is extensive and real, the DMOs should listen to and analyse what locals communicate, especially regarding the communication of the brand and emotional values, to carry out a more coherent destination branding and brand communication.

In addition, the results of the study show the existence of major differences between the brand emotional values communicated by DMOs and those communicated by the locals themselves. Therefore, DMOs need to analyse and consider the communication of brand values by locals, assess them and take into account them for branding strategy. In fact, to avoid inconsistencies in communication, as many studies and authors say [8–11, 19, 39, 40], the branding of tourist destinations should be more participatory, and this poses another challenge for DMOs. Therefore, this work is also in line with these previous studies. Another implication that can be drawn is that DMOs should include the participation of all the locals in the branding process from the outset. Only in this way will they ensure more coherent branding and communication of brand emotional values.

Finally, it is worth pointing out some limitations of our current approach that might lead to lines of future research:

- The system only analyses tweets in English. It could be conceivable to think about the possibility of using automatic translation mechanisms in order to analyse tweets in other languages. This idea is not very far-fetched if only adjectives are analysed. It would also be interesting to make more exhaustive independent studies for English-speaking and non-English-speaking destinations, to assess the possible differences in the usage of adjectives. In this study it may be observed that the DMOs of the English-speaking destinations (London, Edinburgh and Dublin) have the three top positions in the number of adjectives and the number of uses of adjectives; however, when the tweets of locals are considered, then London keeps the first place but Edinburgh and Dublin have very low positions (e.g. 7th and 8th in the number of adjectives).
- We are only taking into account the adjectives present in the tweets, disregarding other information in the text that could be important. In particular, we are not analysing whether the reference to the emotional value is positive or negative. Moreover, we are not studying the possible irony implicit in the tweet, which could be radically changing the meaning of the message. It would also be interesting to study the adverbs present in the tweets.
- Concerning the tweets sent from locals, in the present study it is not possible to ensure that they are actually talking about the destination itself (although a manual analysis of a representative sample indicated that almost 3 out of 4 tweets did indeed refer to local aspects of the destination). Therefore, it would be very interesting to think about possible fliters that could be applied to the dataset to remove tweets that are unrelated to the city.
- We are not analysing other pieces of information which Twitter users employ to transmit feelings and emotions, like images or videos. A picture (for example, a couple kissing in the beach at sunset) can convey a much stronger emotional impact than 140 characters. It might be the case that some of the analysed cities prefer using images to text, and the current system is not able to analyse this information.
- The initial selection of WordNet nouns to refer to each emotional value is quite relevant, and a change of words could lead to an important change in the final results. Moreover, different steps in the analytic process (tweet parsing, part-of-speech tagging, automatic association of nouns to the adjectives appearing in the tweets) certainly may introduce errors in the process.
- The study only analyses how destinations and locals transmit emotional values. One of our immediate research lines is the study of the tweets of visitors to analyze which emotional values are actually perceived in the destination.
- It would also be possible to apply the same methodology to analyze tweets referring to attractions, restaurants, hotels, etc.
